# Genotoxic and Anti-Genotoxic Potential of Hydrosols from Water–Steam Distillation of Oil-Bearing Roses *Rosa centifolia* L. and *Rosa gallica* L. from Bulgaria

**DOI:** 10.3390/ph17050657

**Published:** 2024-05-20

**Authors:** Svetla Gateva, Gabriele Jovtchev, Tsveta Angelova, Tsvetelina Gerasimova, Ana Dobreva, Milka Mileva

**Affiliations:** 1 Institute of Biodiversity and Ecosystem Research, Bulgarian Academy of Sciences, 2 Gagarin Str., 1113 Sofia, Bulgaria; spetkova2002@yahoo.co.uk (S.G.); gjovtchev@yahoo.de (G.J.); angelova_ts@abv.bg (T.A.); cvetij@yahoo.com (T.G.); 2 Institute for Roses and Aromatic Plants, Agricultural Academy, 49 Osvobojdenie Blvd., 6100 Kazanlak, Bulgaria; anadobreva@abv.bg; 3 Institute of Microbiology, Bulgarian Academy of Sciences, 26 Acad. G. Bonchev Str., 1113 Sofia, Bulgaria

**Keywords:** *Rosa centifolia* L. and *Rosa gallica* L. hydrosols, test systems, chromosome aberrations, micronuclei, anti-cytotoxic and anti-genotoxic potential

## Abstract

*Rosa centifolia* L. and *Rosa gallica* L. (Rosaceae) are grown as raw materials for valuable essential oils and hydrosols. There are scarce data about the biological activities and the genoprotective potential of the hydrosols of these roses. The aim of the study was to provide information on their cytotoxic/genotoxic activity and anti-cytotoxic/anti-genotoxic capacity against mutagenic *N*-methyl-*N*′-nitro-*N*-nitrosoguanidine (MNNG). The evaluation was performed using classical tests for chromosomal aberrations and micronuclei in the higher plant *Hordeum vulgare* and human lymphocyte test systems. The experimental schemes included combined hydrosol and mutagen treatment. Both hydrosols (6, 14, 20%) had no cytotoxic effect on barley and showed low genotoxicity in both test systems as the injuries were enhanced to a lesser extent compared to the controls. Lymphocytes were more susceptible than *H. vulgare*. Under the conditions of combined treatment, it was found that the two hydrosols possessed good anti-cytotoxic and anti-genotoxic potential against MNNG. Both rose products exerted genoprotective potential to a similar extent, decreasing the frequencies of aberrations in chromosomes and micronuclei to a significant degree in both types of cells when non-toxic concentrations of hydrosols were applied before MNNG. This was performed both with and without any inter-treatment time. The observed cytoprotective/genoprotective potential suggests that these hydrosols are promising for further application in phytotherapy and medicine.

## 1. Introduction

Cell resistance to DNA-damaging agents is essential for the normal functioning of the cellular genome. There has been growing interest in various plant products and compounds of plant origin that help cells to activate their defence mechanisms. Essential oils [1,2,3,4] and hydrosols and extracts [5,6,7,8] of oleaginous plants have many biological effects due to the wide range of compounds they contain. These compounds contribute to their healing effects, including the protection of the genome against injuries caused by factors of different origin.

The Bulgarian flora is extremely rich in herbs and medicinal plants with significant healing potential. It is believed that Bulgaria is one of the main producers of rose essential oil and other aromatic rose products for use in various spheres of human life. Along with *Rosa damascena* Mill. and *Rosa alba* L., *Rosa centifolia* L. and *Rosa gallica* L. are valuable raw materials used for the production of rose oil and rose hydrosol [9,10,11,12].

Rose hydrosol, also referred to as hydrolat, floral water, or rose water, is obtained during the steam distillation of essential oils or is produced separately. The product contains only a small amount of essential oil. In *R. damascena* Mill., hydrosol is between 10 and 50% of the yield of pure essential oil, which is 0.03–0.04% of the plant matter [3], whereas it is even less in *R. centifolia* L., standing at about 0.01% [13]. The hydrosols include some of the main water-soluble components of essential oils as well as other water-soluble secondary metabolites [14,15,16,17,18,19]. Their chemical composition, on the one hand, depends on the profile of the corresponding essential oil, but on the other, it differs from that because of a lack of hydrocarbons [20]. 

Aromatic products are known to possess valuable biological properties [19,20]. There are many reports on using rose hydrosols in folk medicine for the treatment of cutaneous and mucosal injuries, digestive problems, ophthalmic problems, chronic insomnia, skin and hormonal problems, and neurological problems [21,22,23,24,25]. These products also demonstrate antimicrobial effects [26], anti-inflammatory activity [27], and antimutagenic effects in different cells [28,29]. 

*Rosa centifolia* L. is an old rose species cultivated in Bulgaria, Turkey, Morocco, France, and Italy. It is used for the production of concrete, absolute, and hydrosol. It is known as the province rose, cabbage rose, or hundred-leaved rose. Its essential oil is used in cosmetics, as a fragrance in high perfumery, and as a flavor in confectionery. This rose species has been applied as a therapeutic agent for asthma, high blood pressure, bronchitis, diarrhea, dysmenorrhea, cough, fever, fluid retention, insomnia, arthritis, and urinary infections [30,31,32,33,34]. According to Dobreva et al. [12], the hydrosol from this rose grown in Bulgaria has a higher value of essential oil amounts than species from Iran [12]. *R. centifolia* L. water extract [35] and methanolic extract [36] demonstrate well-expressed antimicrobial activity. The methanolic extract significantly inhibits HIV-1 primary isolates in PM1 cells [37]. Scarce data exist on the cyto- and genoprotective effect of this rose hydrosol. An investigation showed that aqueous extracts of different cultivars reduced the frequency of mutations induced by the mutagen ethyl methane sulfonate in *E. coli* [29].

*Rosa gallica* L. is another plant with industrial and ornamental aromatic uses known since ancient times. It is referred to as the French rose or the Provence rose and is grown not only in Bulgaria but also in other countries of Southern and Eastern Europe, as well as in Armenia, Azerbaijan, and Georgia. The amount of essential oil in the Bulgarian *R. gallica* hydrosol is close to that of *R. centifolia* [12]. This species has applications in the fragrance industry, cosmetics, medicine, and the culinary industry. Its pharmacological properties, however, have been much less studied than those of *R. damascena* Mill. There are some reports of its anti-allergic, antioxidant, skin anti-inflammatory, analgesic, gastroprotective, anti-skin ageing, and antibacterial effects [38,39,40,41,42]. Five types of *R. gallica* var. *aegyptiaca* leaf extracts show good antioxidant and antimicrobial potential [43]. A study [44] has demonstrated that rose petal extracts significantly inhibit the growth of lung and colorectal cancer cell lines. 

**Figure 1:**
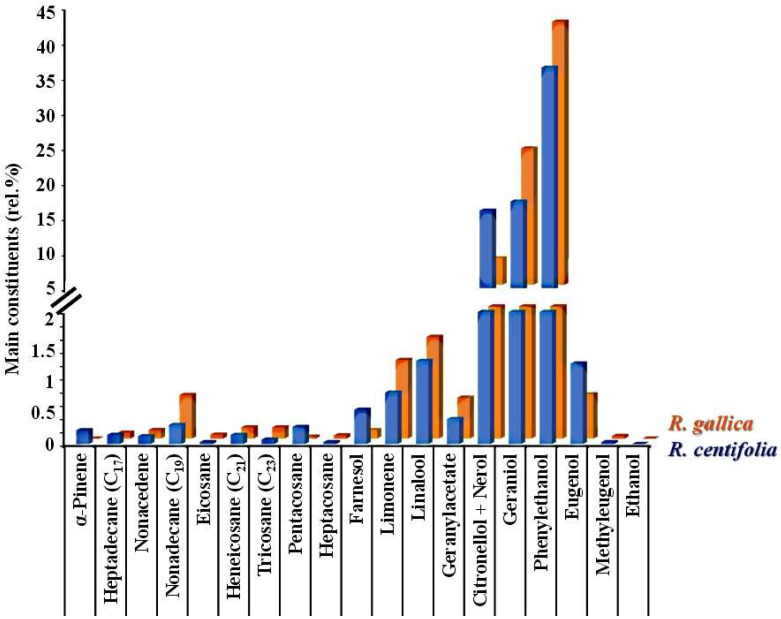
Chemical composition of the *R. centifolia* L. and *R. gallica* L. hydrosols identified by GC-FID/MS analysis described previously by [12].

The chemical composition of both rose products is much less well studied than that of *R. damascena* Mill. [13]. Existing data show that *R. centifolia* and *R. gallica* aqueous and ethanolic extracts [42,45] and the subcritical extracts with 1,1,1,2-tetrafluoroethane (freon R134a) contain monoterpenoids, sesquiterpenoids, triterpenoids, phenylethanoids, phenylpropanoids, aliphatic hydrocarbons, fatty acids, esters, and waxes [46]. For example, the *R. gallica* and *R. centifolia* hydrosols contain various groups of phytochemicals [12], mostly with antioxidant activity (Figure 1).

In recent years, more and more attention has been paid to the possible toxicological and/or genotoxic effects of plant extracts that are used in folk medicine and have valuable therapeutic properties. Despite the therapeutic potential attributed to *R. centifolia* and *R. gallica* hydrosols, there are few studies on their cytotoxicity/genotoxicity and genoprotective potential [47]. These aromatic products contain some valuable chemical compounds with excellent biological effects. This suggests that these rose products would have good cyto- and genoprotective effects. The aim of the present work was to investigate the cytotoxic/genotoxic effect and cytoprotective/genoprotective potential of *R. centifolia* L. and *R. gallica* L. hydrosols against a well-known alkylating mutagen, *N*-methyl-*N*′-nitro-*N*-nitrosoguanidine (MNNG), in a series of broadly used genotoxicity tests and appropriate experimental schemes in two different test systems. Using more than one assay and test systems that are widely used in genotoxic screening is known to enhance the reliability of the obtained data. The results obtained would pave the way for further research on the potential of these rose products to protect the genome against damage caused by alkylating genotoxins.

## 2. Results

### 2.1. Cytotoxic Activity of R. gallica L. and R. centifolia L. hydrosols

**Figure 2:**
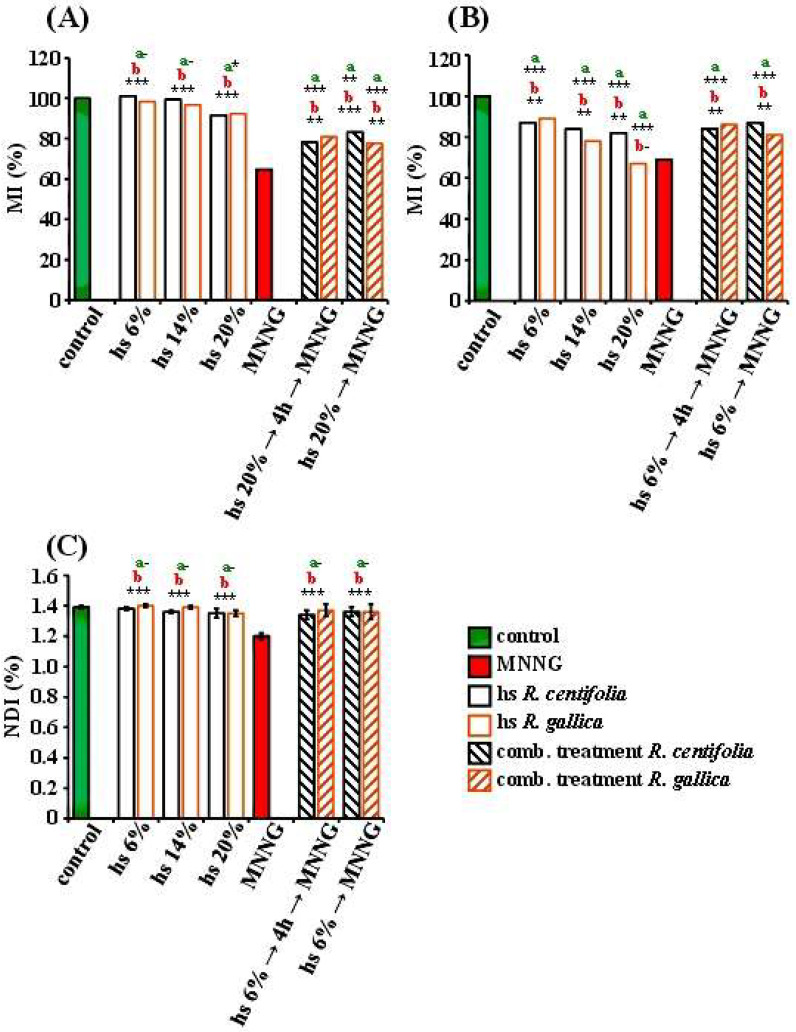
Cytotoxic/anti-cytotoxic activity of *R. gallica* and *R. centifolia* hydrosols (hs), observed after treatment with hydrosol alone and after combined treatment applying schemes with hydrosol and MNNG, with 4 h inter-treatment time between treatments and without any inter-treatment time. These were assessed on the basis of the value of MI in *H. vulgare* (**A**) and human lymphocyte cultures (**B**), and on the basis of the value of NDI in human lymphocyte cultures (**C**). Mitotic activity (MI) was calculated as a percent of the negative control. “a” indicates differences between negative control (untreated variant) and the corresponding treatment variant; “b” indicates differences between positive control (MNNG) and the corresponding treatment variant. The statistical differences were assessed as *** *p* < 0.001, ** *p* <0.01, * *p* < 0.05, dashes (-) *p* > 0.05 non-significantly.

The endpoint for the cytotoxicity of rose hydrosols in both *H. vulgare* and human lymphocytes was the mitotic index (MI) (Figure 2). The applied hydrosol concentrations (6%, 14%) were not cytotoxic for *H. vulgare* meristems; the value of MI calculated for the treated samples was not significantly different from that of the untreated control. Only the highest concentration (20%) of both rose products had low cytotoxic activity (*p* < 0.05). The human lymphocyte cultures were more susceptible to the two hydrosols tested in this study. This was more pronounced for the *R. gallica* hydrosol, where the mitotic activity (i.e., MI values) showed a concentration-dependent trend (*p* < 0.001) (Figure 2A,B). The *R. centifolia* hydrosol had a lower cytotoxic effect than the *R. gallica* hydrosol, but the difference was non-significant. 

The values of MI were lower for both barley and human lymphocytes (*p* < 0.001, *p* < 0.01) than for the alkylating mutagen MNNG. Only treatment with the highest *R. gallica* hydrosol concentration (20%) resulted in MI values close to those of the cells treated with MNNG (50 µg/mL).

The second endpoint for cytotoxicity in lymphocyte cells is the nuclear division index (NDI). It is informative about the proliferative activity of the cells after treatment. In the present study, there were non-significant differences between the values of NDI in both the samples treated with rose hydrosols and the untreated cells. There were also non-significant differences between the tested hydrosols (Figure 2C). The NDIs of the hydrosols were higher than those in the samples with MNNG (*p* < 0.01).

### 2.2. Anti-Cytotoxic Activity of R. gallica L. and R. centifolia L. hydrosols

Two schemes for the combined treatment of hydrosols and MNNG were applied to test the defensive potential of the rose hydrosols in both test systems: the first one was conditioning treatment with hydrosol followed by a challenge with MNNG and 4 h inter-treatment time between the treatments, and the second was treatment with hydrosol and MNNG without any inter-treatment time. The hydrosols’ concentrations used for these treatments were non-toxic or slightly toxic (20% for barley and 6% for lymphocytes). These concentrations were pre-selected based on our previous study. 

In the combined experimental treatment schemes using *R. centifolia* hydrosol, the value of MI was significantly higher (*p* < 0.01, *p* < 0.001) than that of MNNG alone and was close to that of the hydrosol alone (Figure 2A,B). This was observed both in barley and human lymphocytes, regardless of the experimental design. Similar results were obtained for the MI value in the experiments with conditioning treatment with *R. gallica* hydrosol (20% for barley and 6% for lymphocytes), in a challenge with MNNG (50 μg/mL) with 4 h inter-treatment time, and for treatments without inter-treatment time (Figure 2A,B). The mitotic activity, evaluated based on the MI value in these samples, was higher (*p* < 0.01, *p* < 0.001) than that with MNNG alone, and it reached the value of MI with hydrosol in both test systems. As an endpoint for cytotoxicity, the MI value showed that *R. centifolia* and *R. gallica* hydrosols have well-expressed anti-cytotoxic potential.

The anti-cytotoxic effect was also detected using the nuclear division index (NDI) as a cytotoxicity endpoint in human lymphocytes. The values of the NDI after treatment with the same experimental schemes with non-toxic concentrations of (*R. centifolia* and *R. gallica*) hydrosols and a damaging concentration of MNNG were also significantly higher (*p* < 0.01) compared to those of MNNG in both test systems. The NDI values were close to those of the single treatment with the relevant hydrosol concentration. No statistical differences were obtained between the NDI values calculated after the combined treatments for both hydrosols (Figure 2C).

### 2.3. Genotoxic Activity of R. centifolia L. and R. gallica L. hydrosols

The induction of chromosome aberrations (CA) and micronuclei (MI) were used to obtain information about the genotoxicity/anti-genotoxicity of the tested rose hydrosols.

**Figure 3:**
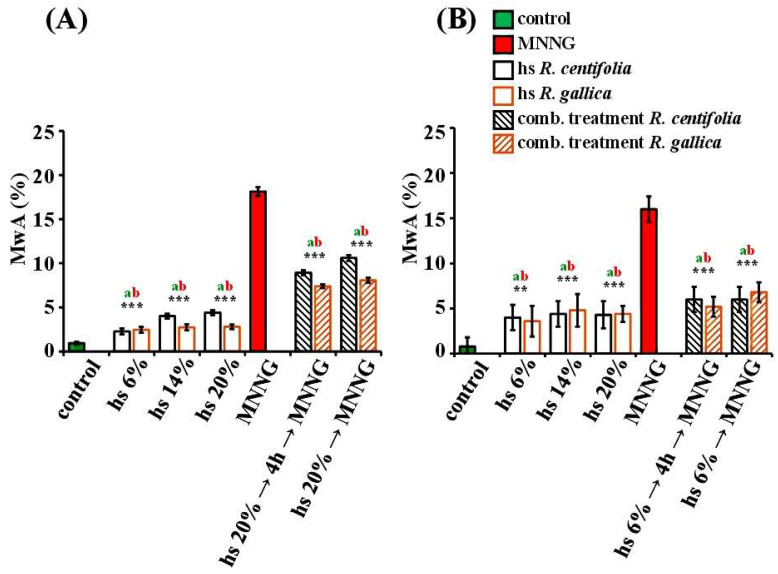
Genotoxic/anti-genotoxic activity of *R. centifolia* and *R. gallica* hydrosols (hs) assessed by the induction of chromosome aberrations after treatment with hydrosol alone and after combined treatment applying schemes with hydrosol and MNNG with 4 h inter-treatment time between treatments, and without any inter-treatment time in *H. vulgare* (**A**) and in human lymphocyte cultures (**B**). “a” indicates differences between negative control (untreated variant) and the corresponding treatment variant; “b” indicates differences between positive control (MNNG) and the corresponding treatment variant. The statistical differences were assessed as *** *p* < 0.001, ** *p* <0.01.

The values of induced CAs after treatment with *R. gallica* and *R. centifolia* hydrosols showed a low but statistically significant genotoxic effect (*p* < 0.001) compared to the negative controls in both test systems (Figure 3A,B). There were no concentration-dependent effects for either hydrosol in plant and human lymphocyte test systems. The *R. gallica* hydrosol showed lower genotoxicity/clastogenicity than the *R. centifolia* hydrosol in *H. vulgare*. The frequency of induced chromosome aberrations ranged from 2.27% ± 0.33 (for 6%) to 4.4% ± 0.29 (for 20%) for *R. centifolia* and from 2.47% ± 0.37 (for 6%) to 2.80% ± 0.26 (for 20%) for *R. gallica*. Both hydrosols had very similar values of clastogenicity in the lymphocyte cells, as there were no significant differences between the frequency of the induced chromosome damage (Figure 3B). They ranged from 4.00% ± 1.7 (for 6%) to 4.30% ± 1.50 (for 20%) for *R. centifolia*, and from 3.60% ± 1.70 (for 6%) to 4.40% ± 0.90 (for 20%) for *R. gallica*. The lymphocyte cultures were more sensitive to the *R. gallica* hydrosol than the barley cells; the value of the induced chromosome injuries was slightly higher (Figure 3B).

The genotoxic activity of both rose hydrosols in the applied concentrations was much lower (*p* < 0.001) than that of the direct mutagen MNNG in both *H. vulgare* and human lymphocytes in vitro (Figure 3B).

**Figure 4:**
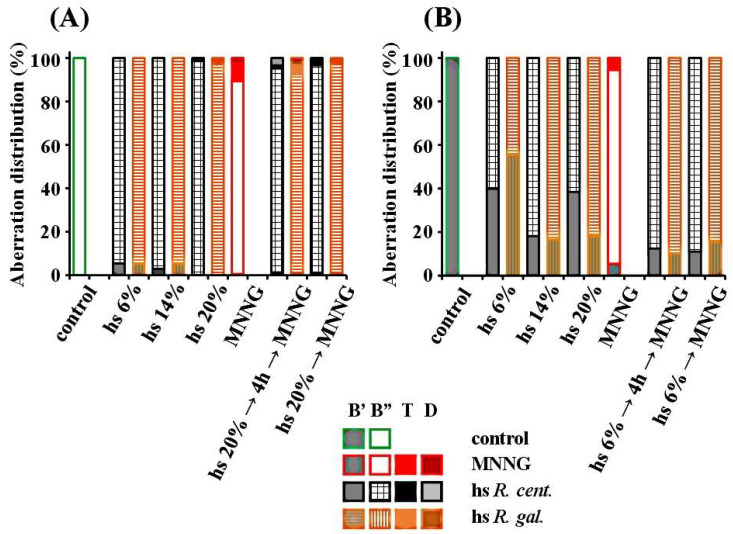
Distribution of the chromosome aberrations observed after treatment with *R. gallica* and *R. centifolia* hydrosols (hs) alone, and with combined treatment using experimental schemes with hydrosols and direct mutagen MNNG, with 4 h inter-treatment time between treatments, and without any inter-treatment time in *H. vulgare* (**A**) and in human lymphocyte cultures (**B**).

The spectrum of induced chromosome aberrations by *R. centifolia* hydrosol in *H. vulgare* included mainly isochromatid breaks (B″), followed by a small percentage of chromatid breaks (B′) and translocations (T), whereas after treatment with *R. gallica* hydrosol, only isochromatid breaks (B″) and chromatid breaks (B′) were detected (Figure 4A). In human lymphocytes, the *R. centifolia* hydrosol predominantly induced isochromatid breaks (B″), followed by chromatid breaks (B′), whereas in some variants treated with *R. gallica* hydrosol (6%), chromatid breaks (B′) were prevalent, followed by isochromatid breaks (B″) (Figure 4B). MNNG induced a wide spectrum of chromosome aberration in both test systems (Figure 4A,B).

**Figure 5:**
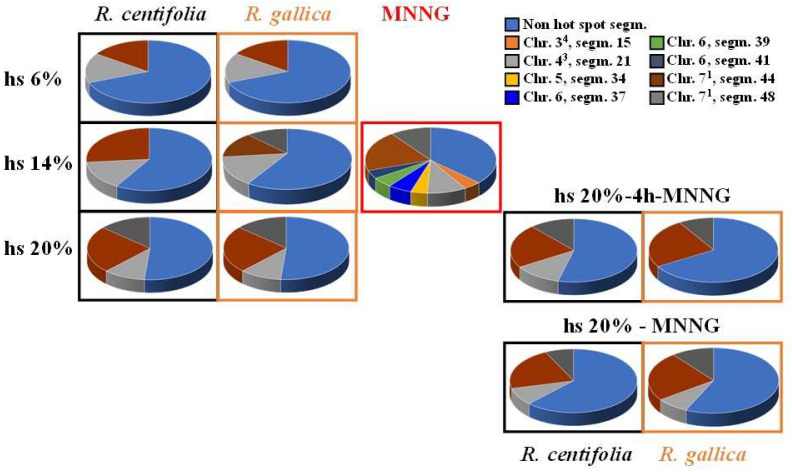
Aberration “hot spots” observed in *H. vulgare* (reconstructed karyotype MK14/2034) meristem root cells after treatment with *R. centifolia* and *R. gallica* hydrosols (hs) alone, and after combined treatment in two experimental schemes with hydrosol and direct mutagen MNNG with 4 h inter-treatment time, and without inter-treatment time.

To assess the sensitivity of different parts of plant chromosomes to the tested rose hydrosols, aberration “hot spots” in *H. vulgare* (reconstructed karyotype MK14/2034) were used. The results showed a concentration dependence (Figure 5). Treatment with 6% *R. centifolia* hydrosol induced 15.4% “hot spots” in segment 21 of chromosome 43 and 15.4% in segment 44 of chromosome 7^1^. Treatment with 14% rose product induced 14.9% “hot spots” in segment 21 of chromosome 4^3^ and 26.5% in segment 44 of chromosome 7^1^. Treatment with 20% hydrosol induced 10.8% “hot spots” in segment 21 of chromosome 4^3^, 24.3% in segment 44 of chromosome 7^1^, and 13.5% in segment 48 of chromosome 7^1^. Overall, the *R. centifolia* hydrosol alone affected only 2 or 3 out of the 48 inspected chromosome segments.

The aberration “hot spots” after *R. gallica* hydrosol treatment were also detected in only two or three chromosome segments (Figure 5). The 6% hydrosol treatment affected segment 44 of chromosome 7^1^ (17.1%) and segment 48 of chromosome 7^1^ (14.3%). Treatment with 14% hydrosol affected segment 21 of chromosome 4^3^ (14.6%), segment 44 of 7^1^ (14.6%), and segment 48 of chromosome 7^1^ (12.2%). Treatment with 20% hydrosol affected segment 21 of chromosome 4^3^ (16.3%), segment 44 of 7^1^ (20.9%), and segment 48 (14.0%) of the same chromosome 7^1^.

MNNG showed significant deviation from the random distribution of isochromatid breaks. Aberrations were observed in “hot spots” in 8 out of all 48 segments of barley (Figure 5).

**Figure 6:**
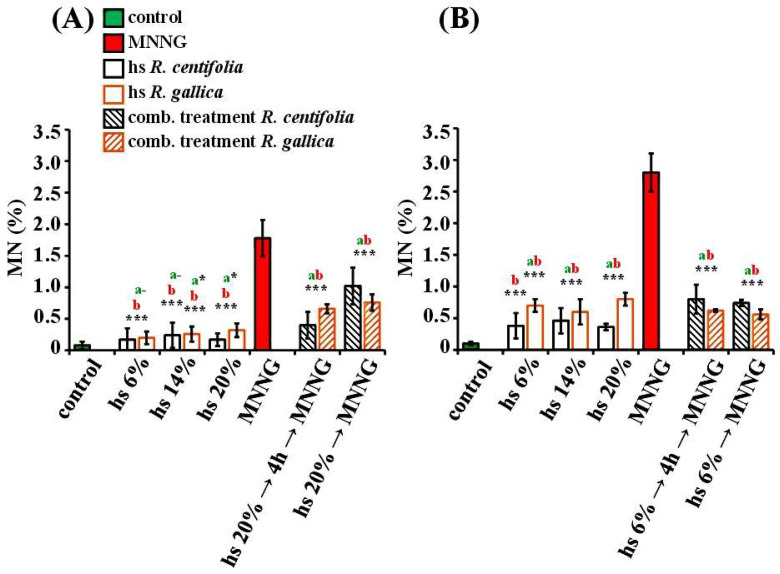
Genotoxic/anti-genotoxic activity of *R. centifolia* and *R. gallica* hydrosols (hs) assessed by the induction of micronuclei (MN) after treatment with hydrosol alone and after combined treatment schemes with hydrosol and MNNG with 4 h inter-treatment time between treatments, and without any intertreatment time in *H. vulgare* (**A**) and in human lymphocyte cultures (**B**). “a” indicates differences between negative control (untreated variant) and the corresponding treatment variant; “b” indicates differences between positive control (MNNG) and the corresponding treatment variant. The statistical differences were assessed as *** *p* < 0.001, * *p* < 0.05, dashes (-) *p* > 0.05 non-significantly.

To obtain more informative results about the genotoxic activity of the tested rose hydrosols, the induction of micronuclei (MN) was also assessed (Figure 6). This endpoint was informative about both chromosome damage and mitotic disturbances. As shown in the figure, the induction of micronuclei was dependent on both the test system and hydrosol. It was interesting to note that MN induction was not dependent on the concentration of the rose hydrosol in *H. vulgare* and in lymphocytes. Barley meristem cells showed the low induction of MN after treatment with *R. centifolia* or *R. gallica* hydrosol (Figure 6A). The frequency of observed injuries caused by *R. centifolia* was in the range from 0.17% ± 0.18 (for 6%) to 0.17% ± 0.10 (for 20%), and for injuries by *R. gallica* hydrosol the frequency ranged from 0.20% ± 0.10 (for 6%) to 0.32% ± 0.11 (for 20%). 

The frequencies of MN induction by both rose hydrosols were statistically significantly higher (*p* < 0.001) than in the control sample in lymphocyte cultures (Figure 6B). In the samples treated with *R. centifolia* hydrosol, the observed MN were in the range from 0.38% ± 0.20 (for the 6% hydrosol) to 0.36% ± 0.05 (for 20% hs). In the cultures treated with *R. gallica*, the MN frequency ranged from 0.70% ± 0.10 (for 6% hs) to 0.80% ± 0.10 (for 20% hs). The *R. gallica* hydrosol induced a higher frequency (*p* < 0.01) of MN than the *R. centifolia* hydrosol (Figure 6B).

In the MN induction assay, the genotoxic effect of both rose hydrosols (in the tested concentrations) was much lower (*p* < 0.001) than that of MNNG (Figure 6A,B).

### 2.4. Anti-Genotoxic Activity of R. centifolia L. and R. gallica L. hydrosols

The anti-genotoxic potential of rose hydrosols was assessed, using the induction of chromosome aberrations as an endpoint. 

The frequency of chromosome aberrations was significantly decreased (*p* < 0.001) after conditioning treatment with *R. centifolia* hydrosol (20% for *H. vulgare* and 6% for lymphocytes) followed by damaging treatment with MNNG (50 μg/mL) and 4 h inter-treatment (8.93% ± 0.28 in barley and 6.00% ± 1.40 in lymphocytes) compared with that calculated after MNNG treatment alone (18.30% ± 0.49 in barley and 16.00% ± 1.40 in lymphocytes) (Figure 3A,B). The genotoxic effect of the mutagen was also reduced (*p* < 0.001) in the experimental scheme of combined treatment without any inter-treatment between the rose hydrosol and the mutagen. The frequency of chromosome aberrations was 10.60% ± 0.32 in barley and 6.00% ± 1.40 in human lymphocytes (Figure 3A,B). The anti-genotoxic effect of *R. centifolia* was manifested in both test systems, regardless of the experimental schemes. The frequency of chromosome aberrations decreased from 1.7 to 2 times in barley and 2.7 times in lymphocyte cultures.

The anti-genotoxic effect of *R. gallica* hydrosol was also demonstrated on the basis of the level of induced chromosome aberrations. In both schemes involving combined treatment with hydrosols and MNNG, there was a significant decrease (*p* < 0.001) in the frequency of the aberrations compared with the MNNG treatment alone (Figure 3A,B). This anti-genotoxic effect was manifested in both test systems. The aberrations after combined treatment with hydrosol (20% for *H. vulgare* and 6% for lymphocytes), followed by 4 h inter-treatment time and challenge with the mutagen (50 μg/mL), were 7.40% ± 0.22 in barley and 5.20% ± 1.10 in lymphocyte cultures. For comparison, those induced by MNNG were 18.30% ± 0.49 in barley and 16.00% ± 1.40 in lymphocytes. Hence, the genotoxic effect of the direct mutagen was reduced nearly twice in *H. vulgare* and three times in human lymphocytes. The reduction in the chromosome damage induced by MNNG in the treatment scheme without inter-treatment time was more than two times that in barley cells and in lymphocytes (Figure 3A,B). The frequencies of the aberrations induced in both variants with combined treatment were similar. 

It is interesting to note that the values of chromosome aberrations were similar in the treatments with the tested rose hydrosols in both the higher plant and lymphocyte cultures. They probably have similar anti-genotoxic potential. 

After both types of combined treatment with the hydrosols (*R. centifolia* or *R. gallica*) and the mutagen, the spectrum of the observed aberrations included isochromatid breaks (B″), followed by a small percentage of chromatid breaks (B′), translocations (T), and deletions (D). In the same schemes of combined treatment in human lymphocytes, only isochromatid breaks (B″) and chromatid breaks (B′) were detected (Figure 4A,B).

There was a significant protective effect against MNNG after conditioning treatment with hydrosol in the variants with 4 h inter-treatment time and without inter-treatment time. Eight “aberration hot spots” were induced by MNNG treatment, whereas only 2 or 3 were obtained after treatments following the schemes with combined treatment, which was almost independent of the experimental design (Figure 5). This was observed using the *R. centifolia* hydrosol and the *R. gallica* hydrosol. The “hot spot” aberrations after conditioning treatment with the *R. centifolia* hydrosol (20%), followed by 4 h inter-treatment time prior to challenge with MNNG, and those in the samples without any time between treatments decreased 1.4 times and 1.7 times, respectively, as compared with those in the MNNG samples. In combined variants with *R. gallica* (20%), 1.9 times and 1.5 times fewer “hot spots” were observed, respectively.

The anti-genotoxic potential of rose hydrosols was also assessed, using micronuclei as an endpoint (Figure 6A,B). The induction of micronuclei decreased (*p* < 0.001) in both *H. vulgare* and human lymphocytes after combined treatment with *R. centifolia* and/or *R. gallica* hydrosols and MNNG compared with the level seen in the samples treated only with mutagen.

In barley, the MN frequency induced by MNNG alone was 1.78% ± 0.29. It was reduced more than four times (0.4% ± 0.21) in the combined variant after conditioning treatment with *R. centifolia* hydrosol (20%) and with 4 h inter-treatment time prior to a challenge with MNNG. The micronuclei in the variant with combined treatment without any inter-treatment time decreased to a smaller extent (1.02% ± 0.29). The rose hydrosol also showed well-expressed anti-genotoxic effects (*p* < 0.001) in the samples with combined treatment with *R. gallica* hydrosol and MNNG, irrespective of the schemes of treatment. The MN frequencies were similar (0.66% ± 0.07 and 0.76% ± 0.13, respectively) (Figure 6A). 

The frequencies of MN were lower in the lymphocyte samples treated with conditioning concentrations of the *R. centifolia* hydrosol (6%), followed by 4 h inter-treatment time and challenges with the mutagen (0.80% ± 0.23), as well as in those without any inter-treatment time (0.74% ± 0.05). The MN induction was reduced by more than three times compared to that in the variants treated with MNNG only (2.80% ± 0.30) (Figure 6B). A similarly well-expressed defence potential was observed in the combined variants using *R. gallica* hydrosol, where the frequencies of injury were 0.62% ± 0.02 and 0.56% ± 0.08, with and without inter-treatment time, respectively.

## 3. Discussion

Research into the biological activity of the hydrosols of various medicinal and ornamental plants has become increasingly important because of their widespread use in traditional and folk medicine. Therefore, it is essential that they are safe, non-toxic, and do not induce alterations in the hereditary material of cells. On the other hand, the study of the protective potential of these hydrosols against various genotoxins is also highly beneficial for healthcare. The use of appropriate assays for analysis in different test systems increases the informative nature of the evaluation.

*R. centifolia* and *R. gallica* are rose species that are well known in many countries. In addition to essential oils, hydrosols can also be derived from them. As there were limited data about the chemical composition of their hydrosols, our previous study analyzed the chemical compounds in these products using roses grown in the Kazanlak region [12].

Through chromatographic analysis, 21 chemical compounds were detected in both *R. centifolia* and *R. gallica* hydrosols [12]. They belonged to several main groups: monoterpene oxygenated derivatives, phenol derivatives, monoterpene hydrocarbons, and hydrocarbons. The high amount of oxygenated compounds in these rose products is due to their higher solubility in water. Hydrocarbons, on the other hand, are less soluble and therefore are present in small quantities. The main chemical compounds in hydrosols are phenylethyl alcohol (36.61% in *R. centifolia* and 42.47% in *R. gallica*), geraniol (17.55% in *R. centifolia* and 24.42% in *R. gallica*), citronellol + nerol (16.25% in *R. centifolia* and 8.80% in *R. gallica*) and linalool, limonene, eugenol, and geranyl acetate. Their presence ranges from 0.38% to 1.54% in the two rose species. These compounds are present in both rose hydrosols, but their quantity depends on the rose species from which the rose product is derived. This suggests some differences in the biological activity of the rose hydrosols.

Despite the use of *R. centifolia* and *R. gallica* hydrosols in different spheres of human life, including cosmetics, folk medicine, and the culinary industry, there is scarce information about their cytotoxic and genotoxic effects. In the present study, as an endpoint for cytotoxicity, the MI assay showed that both hydrosols in concentrations of 6% -20% do not induce any cytotoxic effects in barley meristem cells. The lymphocyte cells were more susceptible to these rose products than barley, as the cytotoxic effect increased in a concentration-dependent trend. The presence of cell walls in plants makes their cells less vulnerable to various injuries than lymphocyte cells in vitro due to their different permeabilities to chemical compounds. The presence of some main chemical compounds in the tested hydrosols may explain their observed cytotoxic effects in lymphocytes. The chemical constituents identified in higher quantities in our previous study [12] included geraniol and nerol. They belong to the group of monoterpene oxygenated derivatives, and manifest cytotoxic activity in a concentration-dependent manner in various cell types [48,49]. Geraniol treatment at concentrations of 10–100 µg/mL causes no high cytotoxicity in human lymphocytes, whereas it does not inhibit cell viability in barley cells [49]. Coêlho et al. [50] reported that nerol demonstrated significant cytotoxic effects in *Artemia salina* and mouse erythrocytes in concentrations ranging from 31.25 to 500 μg/mL. Other researchers [51] have also found that the presence of higher amounts of monoterpenes or even other unidentified compounds in rosemary and sage hydrosols could be responsible for their toxicity. In the present study, it is interesting to note that cell viability was not affected by hydrosol treatment at the applied concentrations, as assessed by NDI. This was an indicator that most of the lymphocyte cells had entered and completed one or even more division cycles after hydrosol treatment and were not seriously damaged. If lymphocytes have extensive chromosome damage, they either die before cell division or are less likely to enter this phase [52]. A similar good proliferative rate (80% or more) was observed in normal lung Mlg and WI-38 cells treated with various concentrations of *R. gallica* petal extract [44].

In the present study, the genotoxic effect of the rose hydrosols (6–20%) was assessed using two widely applied tests for the induction of chromosome aberrations and micronuclei. Both hydrosols did not show high genotoxicity, but the frequencies of aberrations and micronuclei were higher compared with the non-treated plant cells and cultured human lymphocytes. The DNA of cultured human lymphocytes was more susceptible to hydrosols than that of *H. vulgare* cells. Previous studies demonstrated that treatment with *R. alba* L. and *R. damascena* Mill. hydrosols (3–20%) also increased the frequency of chromosome injuries and micronuclei to a low extent in the used test systems [53,54]. The observed effect is probably due to the chemical compounds in the hydrosols that can interact with both *H. vulgare* and human lymphocyte DNA. For example, there are large amounts of monoterpenoids and phenyl derivatives. A previous study reported that the monoterpenoid geraniol and monoterpene aldehyde citral A increased the percentage of migrated DNA in the comet tail of *H. vulgare* meristem cells [55]. Geraniol increased chromosome aberrations and micronuclei in human lymphocytes as well [49]. Silva et al. [56] reported significant DNA damage and chromosomal mutations after nerol application in a medium, and they found high concentrations in human peripheral blood mononuclear cells and HepG2/C3A-cultured cells. It was proposed that the genotoxic effect of these compounds is due to the presence of an oxygen-related hydroxyl group and carbon double bond that can increase the structure’s electronegativity and interfere with metabolic processes involving the electron transfer that leads to interactions with DNA [57]. The observed genotoxic effect of the tested hydrosols can be due not only to a single chemical compound but also to the synergistic action of the chemical compounds present in rose hydrosols, in turn affecting DNA and the mitotic apparatus. A similar effect was observed for aqueous and hydro-methanol extracts of four different plants in mice bone marrow cells [6].

The lack of a clear difference between the genotoxic effect of *R. centifolia* and *R. gallica* hydrosols, assessed both by frequencies of chromosome aberrations and micronuclei in plants and human lymphocyte test systems, is probably due to the presence of the same chemical substances in similar amounts in these rose products [12].

On the other hand, the chemical composition of *R. centifolia* and *R. gallica* hydrosols obtained in the previous study included the secondary metabolites geraniol, nerol, limonene, linalool, and citronellol [12]. They have well-expressed pharmacological and antioxidant activities [50,58,59,60,61,62,63,64]. Many studies reported that antioxidant compounds also have antimutagenic, anti-genotoxic and anti-cancerogenic effects [7,65,66]. This fact challenged us to study the anti-cytotoxic and anti-genotoxic potential of rose hydrosols against the direct mutagen MNNG. Investigating the defence potential of hydrosols is important in terms of exploring their activity in preventing mutations induced by various genotoxins.

MNNG is a well-known monofunctional alkylating experimental mutagen. It can induce DNA double-strand breaks, intra-strand, and inter-strand crosslinks in various cells [67,68]. Chromosome aberrations, sister chromatid exchanges, DNA strand breaks, and unscheduled DNA synthesis were reported after treatment with MNNG [69]. MNNG reacts with the O^6^ atom of guanine and forms an O^6^alkylG adduct that is highly mutagenic [70,71]. This DNA base can miscode and direct the introduction of the incorrect pyrimidine as its complementary base. If the *N*-alkylated purines are not removed by base excision repair (BER), they cause chromosome aberrations. In addition to its alkylating activity, MNNG can trigger ROS production, resulting in enhanced DNA damage and PARP-1 activation in mouse embryonic fibroblasts [72].

The results show that both rose hydrosols manifest anti-cytotoxic and anti-genotoxic effects in plant cells and in human lymphocytes against MNNG. The cytotoxic effect of MNNG was alleviated, as assessed by cytotoxicity endpoints (MI and NDI). The values of the endpoints for cytotoxicity were significantly decreased compared with those of MNNG alone. This effect was observed irrespective of the experimental schemes of treatment, and occurred with or without inter-treatment time between the hydrosols and the mutagen.

The tested hydrosols from *R. centifolia* and *R. gallica*, respectively, decrease the damaging effect of MNNG and demonstrate well-expressed anti-genotoxic potential. This protective potential was observed in both test systems, regardless of the experimental treatment scheme. Both endpoints for genotoxicity, the frequencies of chromosome aberrations and micronuclei, were reduced when non-toxic concentrations (6% for lymphocytes and 20% for *H. vulgare*) of hydrosol (*R. centifolia* or *R. gallica*) were applied before MNNG, with and without any inter-treatment time. It is interesting to note that the number of DNA damage-sensitive fragments, known as “aberration hot spots”, detected in *H. vulgare* meristems was reduced compared with that induced by the mutagen alone. In addition, the affected sections are mainly located in regions that are less important for maintaining the functionality of the chromosome.

The results agree with our previous study, where the application of *R. damascena* hydrosol in non-toxic concentrations (6% in lymphocyte cultures and 20% in barley) also demonstrated cyto- and genoprotective effects against the alkylating mutagen MNNG [54]. Similar DNA-protective potential was also reported for the *R. alba* essential oil [2].

Both hydrosols showed similar anti-genotoxic potential, as the frequencies of chromosome aberrations and micronuclei were similar. This could be attributed to the presence of the same phytochemicals (secondary metabolites), which are present in comparable amounts in both rose products. Examples include monoterpene oxygenated derivatives, as well as phenol compounds that are present in high quantities in these hydrosols [12]. Geraniol, citronellol, nerol, and linalool are present in high amounts in both hydrosols; hence, they can participate in the protective potential of the tested hydrosols. According to many studies, monoterpenes have a leading role in the antioxidant properties of hydrosols. Geraniol and citronellol can inhibit lipid peroxidation in rat brain homogenates [61]. Nerol can decrease the liver damage induced by carbon-tetrachloride (CCl_4_) [73]. These compounds with good antioxidant and antiradical scavenging activity [19] can suppress DNA injuries. A previous study found that geraniol manifests well-expressed anti-cytotoxic and anti-genotoxic potential against MNNG in both barley and lymphocyte cells [49]. Hence, in addition to the cyto- and genotoxic activity, geraniol and nerol demonstrate protective potential, which indicates their dual role. Some studies reported that certain phytochemicals can both induce and prevent DNA damage [74]. A study with aqueous and hydro-methanol extracts of other plants reported a dual effect of the extracts [6]. The authors demonstrated the genotoxic and antigenotoxic effects of the extracts against the monofunctional alkylating agent MMS.

The anti-cytotoxic/anti-genotoxic effect of the tested *R. centifolia* and *R. gallica* hydrosols is not only due to a single phytochemical, but also to a combination of several compounds that act synergistically in the rose products. According to Briskin et al. [75], the protective action or harmful effects of different natural plant products depend on the combined action of their phytochemicals.

The genoprotective effect of the tested rose hydrosols against MNNG probably includes more than one defence mechanism. It may also include the direct damage reversal repair of O^6^alkylG DNA adducts by the enzyme O^6^-methylguanine-DNA methyltransferase (MGMT). Another potential mechanism is ROS scavenging, e.g., blocking the binding sites of the reactive species and/or antioxidant activity of the phytochemicals in the hydrosols. Performing more studies using other test systems and methods would contribute to a better understanding of this defence mechanism.

The evaluation of the anti-cytotoxic/anti-genotoxic potential of the hydrosols can be helpful for utilizing their therapeutic potential and performing further investigations on the pharmacological properties of these plant products.

## 4. Materials and Methods

### 4.1. Chemicals

An RPMI 1640 lymphocyte culture medium was purchased from Sigma-Aldrich (Steinheim, Germany); we obtained fetal calf serum from Sigma–Aldrich (Sao Paulo, Brazil); we received phytohemagglutinin (PHA) and cytochalasin-B from Sigma-Aldrich (Jerusalem, Israel); and we obtained KCl and acid aceticum glaciale from Sigma-Aldrich Chemie GmbH, Merck (Steinheim, Germany). Solutions of 0.9% NaCl and gentamycin 40 mg were provided from Sopharmacy (Sofia, Bulgaria), and a Giemsa stain solution was obtained from Sigma-Aldrich (Buchs, Switzerland). The experimental mutagen *N*-methyl-*N*′-nitro-*N*-nitrosoguanidine (MNNG) (CAS-Nr.: 70-25-7) was obtained from Fluka-AG (Buchs, Switzerland).

### 4.2. Preparation of R. gallica L. and R. centifolia L. hydrosols

Fresh flowers of *R. gallica* and *R. centifolia* L. were collected from private plantations in the Kazanlak Valley, Bulgaria, during the flowering period of 2020, early in the morning (6:00–8:00 a.m.), as described by Dobreva et al. (2023) [12]. The authenticity of the plants was confirmed by DSc. Nelly Grozeva from Trakia University, Stara Zagora (BG). The voucher number for *R. gallica* is SOM 178 485 and for *R. centifolia* it is SOM 178 486. These plants were deposited in the IBER-BAS herbarium. The hydrosols were derived using established technology in Bulgaria. The parameters for distillation using a semi-industrial processing line were as follows: 8 kg of fresh rose flowers for charge and hydro modules (flowers and water) in a ratio of 1:6, with a duration of 3 h at a distillate temperature of 28–30 °C. The first hydrosol obtained from this process was redistilled at a very low speed until an amount equal to the inserted material was obtained using the same apparatus at the same temperature. The rose products were stored at 4 °C in darkness and then sealed in sterilized bottles for further use [12].

### 4.3. Test Systems

We used two different types of test systems, widely applied in genotoxic screening, that operate on different hierarchical levels: we used the higher plant *Hordeum vulgare* and human lymphocytes in vitro to study the cytotoxic/genotoxic activity and anti-cytotoxic/anti-genotoxic effects of the rose hydrosols. Tests for the induction of chromosome aberrations (CA) and micronuclei (MN) were used in both test systems. Specifically, experimental schemes with combined treatment, well established in our previous studies, were applied.

#### 4.3.1. Preparation of Plant Test System and Design of Treatment

**Figure 7:**
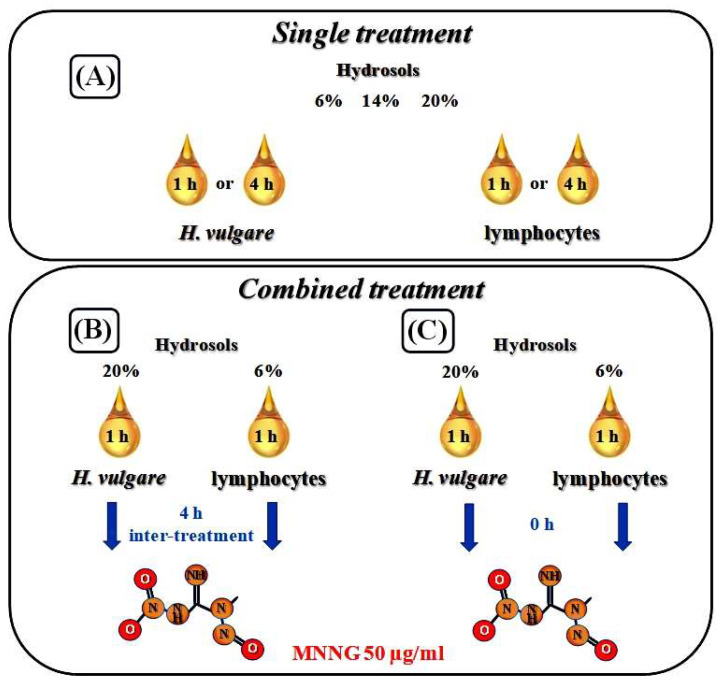
Schemes of treatment: (**A**) single treatment with different concentrations of *R. centifolia* and/or *R. gallica* hydrosol, (**B**) combined treatment with hydrosol and MNNG with 4 h inter-treatment time, and (**C**) combined treatment without any time between treatments in *H. vulgare* and human lymphocytes.

A reconstructed karyotype (MK14/2034) of *H. vulgare* (barley) was used for the current experiments [76]. Presoaked seeds of barley (1 h tap water) were germinated for 17 h in Petri dishes on moist filter paper at 24 °C. After the germination of the barley seeds, part of the root tip meristem of *H. vulgare* was treated with *R. gallica* and/or *R. centifolia* hydrosol for 4 h in concentrations of 6%, 14%, and 20% in order to test the cytotoxic and genotoxic activity of each rose product. To test the defence potential of the hydrosols, the meristems were treated by applying experimental schemes with combined treatments. Some of the barley root tips were subjected to conditioning treatment (60 min) with a non-toxic and low toxic concentration of hydrosol 20% (preselected), followed by 4 h inter-treatment time, and after that we applied a damaging concentration of the direct-acting alkylating mutagen MNNG (50 µg/mL). Another part of the root meristem underwent the same treatment, but without any inter-treatment time (see Figure 7). After each treatment, the barley root tips were rinsed in distilled water. For chromosome aberrations, after the time for recovery (18, 21, 24, 27, and 30 h), the root meristems were treated with 0.025% colchicine in a saturated solution of a-bromonaphthalene (2 h) followed, by fixation in ethanol–glacial acetic acid (3:1). Feulgen staining (Schiff’s reagent, 1 h) was carried out after hydrolyzation in 1N HCl at 60 °C (9 min). After that, the roots were macerated in 4% pectinase, and cut root tips meristems were squashed onto slides. Untreated root tip meristems were used as a negative control. The positive control, MNNG, was dissolved in distilled water to create a stock solution. From this, a working concentration of 50 µg/mL was prepared. For scoring MN, colchicine treatment was omitted, and the root tip meristems were fixed after 30 h recovery time [77].

#### 4.3.2. Preparation of Human Lymphocytes In Vitro and Design of Treatment

The lymphocyte cultures with a cell density of 1 × 10^6^ mol/L were prepared from peripheral venous blood of healthy donors. These subjects were non-smoking/non-drinking, aged between 33 and 40 years (men and women), had no recent history of illness, and had not experienced any contact with medications and mutagens. All procedures in this study were carried out in accordance with the Declaration of Helsinki and after obtaining the necessary ethical approval of the Ethics Commission and Academic Unity of the Institute of Biodiversity and Ecosystem Research (protocol dated 18 February 2022). All donors signed written informed consent forms. Each lymphocyte culture, containing 0.5 mL of lymphocyte suspension, 3.5 mL of RPMI 1640 medium, 12% fetal calf serum, 40 mg/mL gentamycin, and 0.1% phytohemagglutinin (PHA), was cultured at 37 °C.

The method of Evans [78] was applied in experiments for the induction of chromosome aberrations (CA). To test the cytotoxic and genotoxic effects of the rose hydrosols, lymphocyte cells were treated (4 h) with *R. centifolia* and/or *R. gallica* hydrosol in concentrations of 6%, 14%, and 20% at 18 h after PHA stimulation (G1). To study the anti-cytotoxic/anti-genotoxic potential of both rose products, part of the lymphocytes was subjected to conditioning treatment (60 min) with a non-toxic (preselected) concentration of hydrosol (6%), followed by 4 h inter-treatment time and a challenge with (50 μg/mL) of MNNG (60 min). Another part of the cells was treated with hydrosol. This was followed immediately by mutagen treatment (50 μg/mL) without any inter-treatment time (see Figure 7). After each treatment, the cells were washed in a fresh medium and kept at 37 °C until harvesting. At the 72nd hour after PHA stimulation, 0.02% colchicine was added to each culture, followed by hypotonization with 0.56% KCl, fixation in methanol–acetic acid (3:1, *v*/*v*), and stained in 2% Giemsa solution. Non-treated cells were used as a negative control, and treatment with MNNG (50 μg/mL) served as a positive control.

For the micronuclei induction (MN) assay, in order to stop cytokinesis, cytochalasin-B (6 μg/mL) was added to each culture at the 44th hour after PHA stimulation, which was performed according to the cytokinesis-block micronucleus assay (CBMN) [79]. At the 24th hour after Cyt-B treatment, the cells were centrifuged, hypotonized with 0.56% KCl, and fixed in methanol–acetic acid (3:1). After centrifugation, the cell suspension was dropped onto clean slides and stained in 2% Giemsa.

### 4.4. Endpoints

#### 4.4.1. For Cytotoxicity

The endpoints for cytotoxicity were mitotic index (MI) and nuclear division index (NDI). They were calculated as follows.

The value of the mitotic index (MI) was calculated for each experimental variant in both test systems using the test for chromosome aberrations. It was calculated by the formula MI = A/1000, where A represents the number of metaphases.

In human lymphocyte cultures, cytotoxicity was assessed with one more endpoint, nuclear division index (NDI), using the test for the induction of micronuclei. It was calculated using the following formula, NDI = (N1 + 2N2 + 3N3 + 4N4)/N, where N1–N4 represents the number of cells with 1–4 nuclei and N is the total number of scored cells.

#### 4.4.2. For Genotoxicity

The endpoints for genotoxicity were both the induction of chromosome aberrations (CA) and micronuclei.

For chromosome aberrations, the percentage of metaphases with aberrations (MwA% ± SD) was calculated. More than 3000 cells were determined to be in both test systems for each experimental variant. Chromatid breaks (B′), isochromatid breaks (B″), chromatid translocations (T), and intercalary deletions (D) were determined.

‘Aberration hot spots’ were calculated in barley chromosomes (reconstructed barley karyotype MK14/2034) to obtain detailed information about DNA segments that are susceptible to the tested rose products and the experimental mutagen, respectively. Since in karyotype MK 14/2034, in contrast to the standard barley karyotype, all chromosome pairs differ from each other, it is possible to display locus-specific aberrations in metaphase chromosomes. In addition to the visible differences between the individual pairs of chromosomes, they were divided into 48 segments of approximately the same size. The protocol of Rieger et al. [80] and Jovtchev et al. [81] was used to calculate the aberration frequency and its accumulation in the so-called ‘hot spots’.

The percentage of micronuclei (MN% ± SD) was determined. For each experimental variant, 6000 cells were analyzed to assess the presence of MN and spindle defects, which later form MN too.

### 4.5. Statistical Analysis

One-way ANOVA with two-tailed Fisher’s exact test (Microsoft Excel 2010) was used for statistical analysis. Differences were considered statistically significant at the levels of * *p* < 0.05, ** *p* < 0.01, and *** *p* < 0.001. All experiments were repeated three times.

## 5. Conclusions

*R. centifolia* L. and *R. gallica* L. *hydrosols* have weak genotoxic effects in *H. vulgare* and human lymphocyte cells, with the latter being more susceptible to their impact. Both rose products, when applied in non-toxic concentrations, can ameliorate the cytotoxic/genotoxic effect of the mutagen MNNG in schemes involving combined treatment with the mutagen, irrespective of the test systems used. The protective anti-genotoxic potential was manifested to a similar extent by both rose products, decreasing the frequencies of chromosome aberrations and micronuclei. The obtained data showed that the combination of the phytochemicals present in the extracts contributed to a decrease in the DNA damage induced by the genotoxin.

## Data Availability

The data that support the findings from this study are available from the corresponding author upon reasonable request.
